# 299. Consequences of Delaying Surgical Intervention in Patients with Native Joint Septic Arthritis

**DOI:** 10.1093/ofid/ofaf695.101

**Published:** 2026-01-11

**Authors:** Takahiro Matsuo, Ryan B, Khodadadi, Brian Lahr, Omar M Abu Saleh, Pansachee Damronglerd, Jack W McHugh, Said El Zein, Gina A Suh, Aaron J Tande

**Affiliations:** Mayo Clinic, Rochester, MN; Vanderbilt University Medical Center, Nashville, Tennessee; Mayo Clinic, Rochester, MN; Mayo Clinic, Rochester, MN; Faculty of Medicine Thammasat University, Rochester, Minnesota; Mayo Clinic, Rochester, MN; Mayo Clinic, Rochester, MN; Mayo Clinic, Rochester, MN; Mayo Clinic, Rochester, MN

## Abstract

**Background:**

Native joint septic arthritis (NJSA) is a serious musculoskeletal infection requiring timely surgical intervention to prevent long-term morbidity. Although current guidelines recommend early surgical debridement, supporting data, particularly on long-term outcomes, remain scarce. Given this, we evaluated the association between surgical timing and 1-year clinical outcomes using the Desirability of Outcome Ranking (DOOR) framework, applied for the first time in NJSA.

**Table 1.**
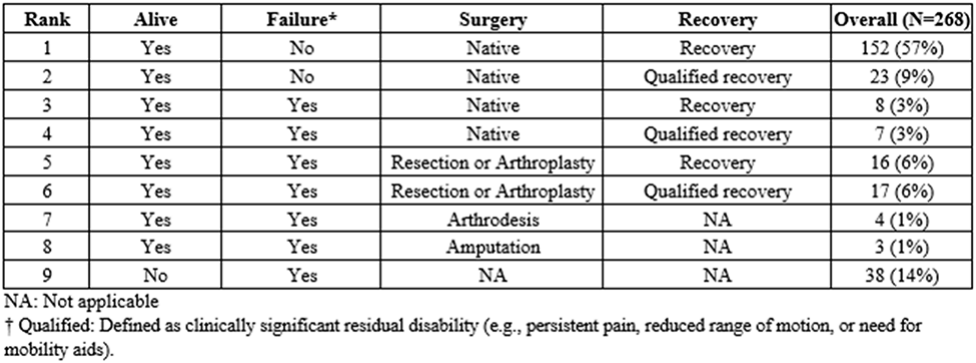
Desirability of Outcome Ranking (DOOR) ordinal scale

**Table 2.**
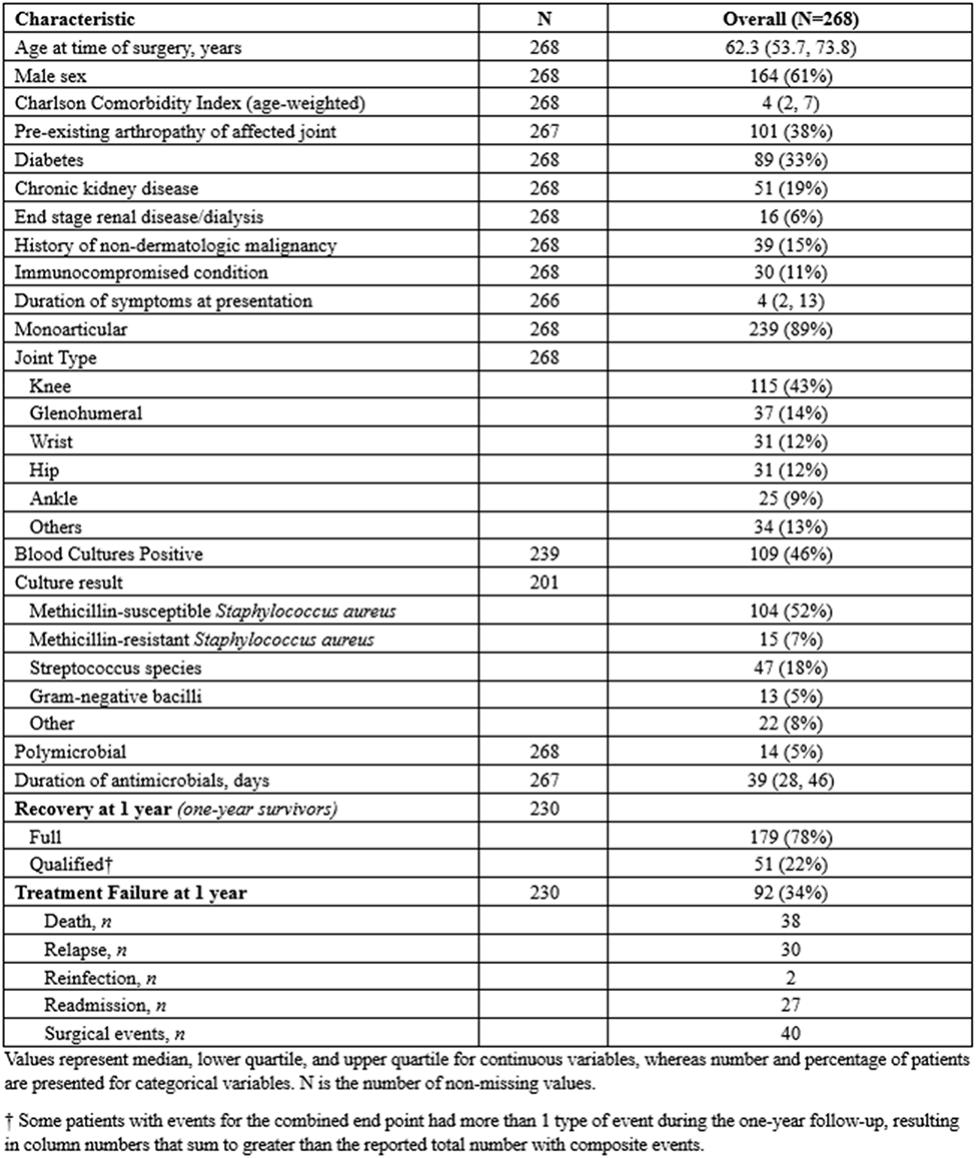
Baseline demographic, clinical characteristics, and treatment outcomes

**Methods:**

We conducted a retrospective multicenter cohort study of adults (≥18 years) with NJSA who underwent surgery across Mayo Clinic campuses between 2012 and 2021. Clinical outcomes at one year were assessed using a 9-level DOOR scale, incorporating survival, treatment failure (relapse, reinfection, readmission, or significant surgical events), and joint recovery (full vs qualified) (Table 1). Time to surgery from hospital admission was analyzed both as a continuous variable and as categories: < 1, 1–2, or ≥3 days.

**Table 3.**
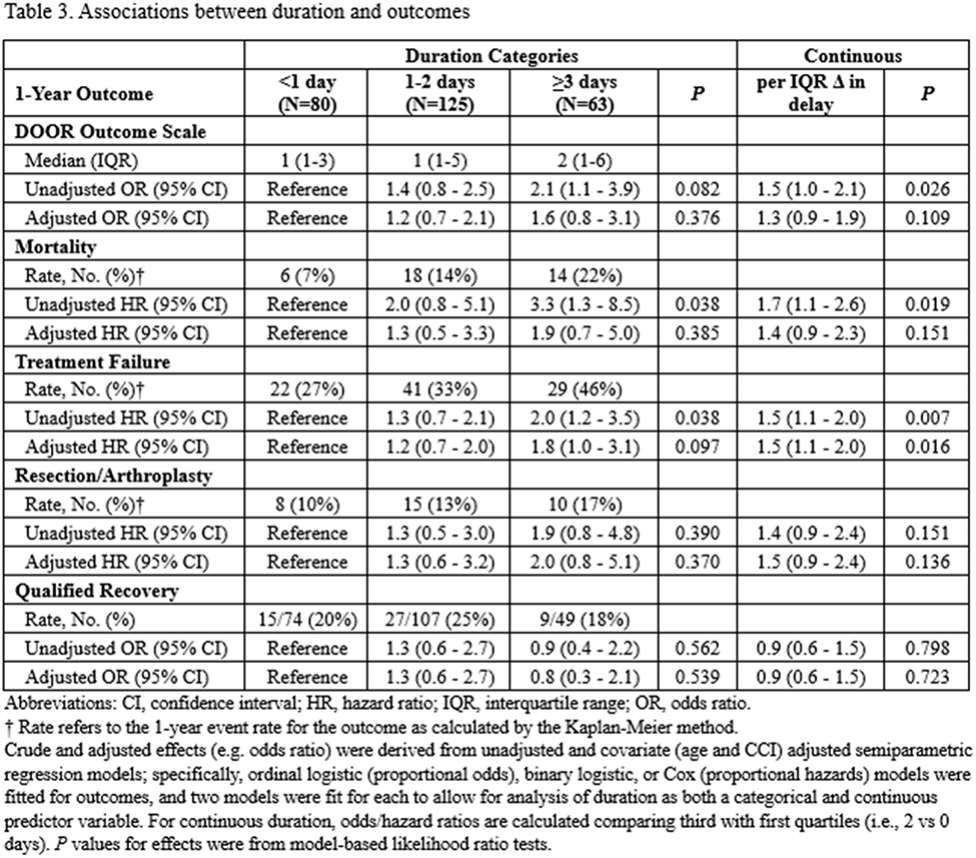
Associations between duration and outcomes

**Figure 1.**
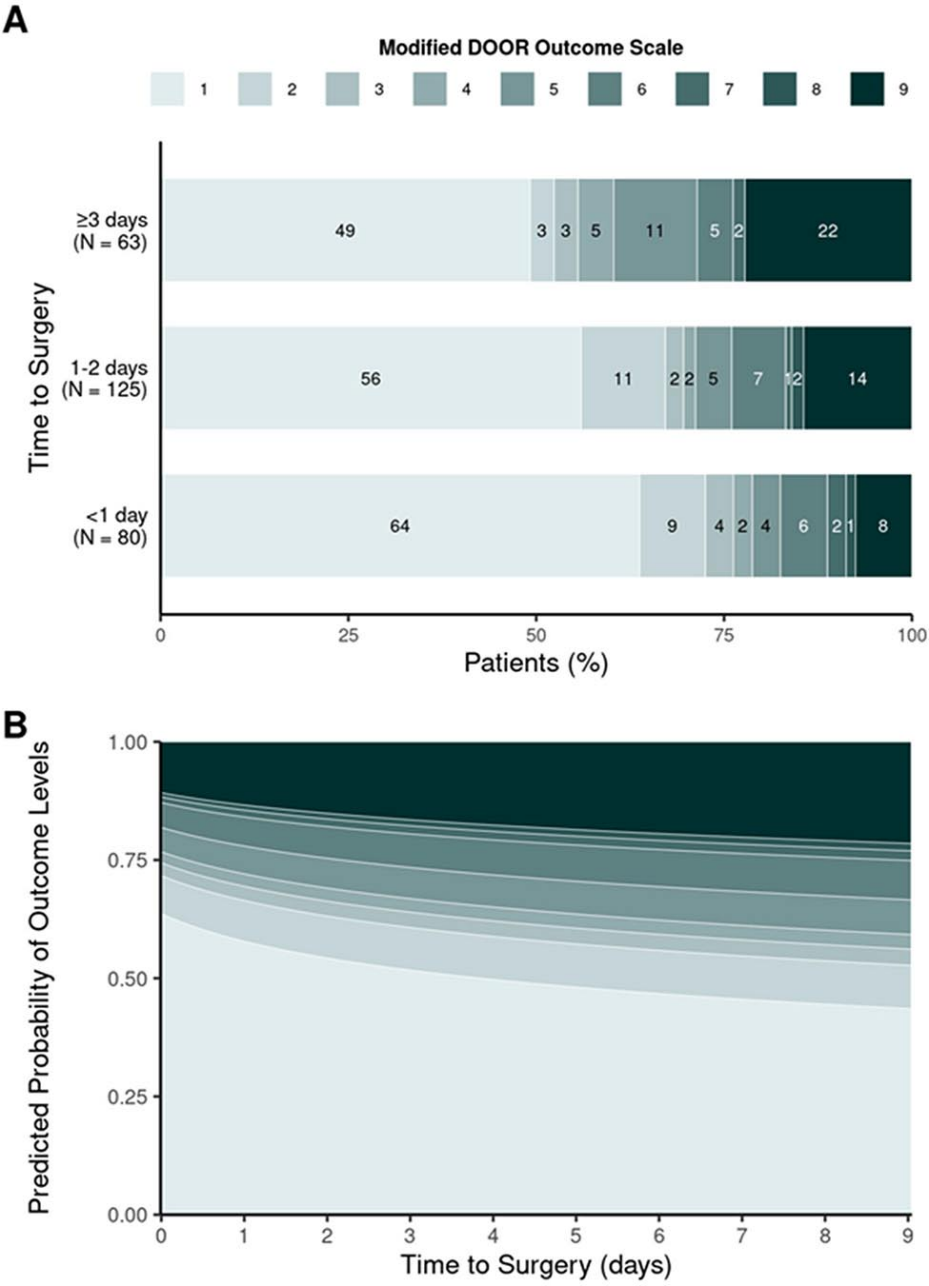
Association Between Time to Surgery and DOOR Scores in Native Joint Septic Arthritis. A) Distribution of Modified DOOR Scores by Surgical Timing. B) Modeled Probability of DOOR Scores by Time to Surgery.

**Results:**

Among 268 patients (median age 62 years, 61% male), 30% underwent surgery < 1 day from admission, 47% within 1–2 days, and 24% at ≥3 days. At 1 year, 57% achieved full recovery without unfavorable events (DOOR score 1), while treatment failure occurred in 34% (Table 2). In unadjusted analyses, longer surgical delay was significantly associated with higher DOOR scores (per IQR increase [from 0 to 2 days], OR: 1.5; 95% CI: 1.0–2.1; p = 0.026) and with increased 1-year mortality (HR: 1.7; 95% CI: 1.1–2.6; p = 0.019) and treatment failure (HR: 1.5; 95% CI: 1.1–2.0; p = 0.007). Similar results were obtained when delay was used as a categorical 3-level variable, with the highest risk observed in patients undergoing surgery after ≥3 days. After adjusting for age and Charlson index, only the association between surgical delay and treatment failure remained statistically significant (HR: 1.5; 95% CI, 1.1–2.0; p=0.016) (Table 3, Figure 1).

**Conclusion:**

Delayed surgical intervention for management of NJSA was independently associated with higher risk of treatment failure at 1 year. These findings reinforce current guideline recommendations for timely operative management and further underscore the potential of the DOOR framework to capture clinically meaningful, multidimensional outcomes in NJSA.

**Disclosures:**

All Authors: No reported disclosures

